# Structure-function analysis of CNGA3-associated achromatopsia patient variants complements clinical genomics in pathogenicity determination

**DOI:** 10.1186/s13023-025-03792-3

**Published:** 2025-05-30

**Authors:** Ditte K. Rasmussen, Young Joo Sun, Joel A. Franco, Aarushi Kumar, Jennifer T. Vu, Alexander G. Bassuk, Vinit B. Mahajan

**Affiliations:** 1 Molecular Surgery Laboratory, Stanford University, Palo Alto, CA USA; 2 Department of Ophthalmology, Byers Eye Institute, Stanford University, Palo Alto, CA 94304 USA; 3 Department of Biomedicine, Aarhus University, Aarhus, Denmark; 4 Departments of Pediatrics and Neurology and The Iowa Neuroscience Institute (INI), University of Iowa, Iowa City, IA USA; 5 Veterans Affairs Palo Alto Health Care System, Palo Alto, CA USA

**Keywords:** Achromatopsia, CNGA3, Cyclic nucleotide-gated ion channel, In silico analysis, Variant classification, Color vision defects, Genetic testing, Molecular structure, Genetic therapy

## Abstract

**Background:**

Achromatopsia is an autosomal recessive genetic disease, and 95% of achromatopsia patients carry pathogenic mutations in the *CNGA3* and *CNGB3* genes. Once translated, these genes function together by forming a cone photoreceptor CNG channel protein complex.

**Results:**

There are 150 *CNGA3* missense variants reported in achromatopsia patients, but the pathogenicity of 103 variants remains unknown due to inconclusive genetic information. Here, we present clinical features of a novel *CNGA3* variant in an achromatopsia patient and demonstrate its pathogenicity by a three-dimensional (3D) proteoform-based structure-function analysis. We first identified six proteotypic groups using 47 pathogenic missense variants with distinctive functional consequences by mapping their spatial proximity in a 3D protein structure. This meta-analysis was further applied to 103 missense variants of unknown significance (VUS) found in patients with achromatopsia. Strikingly, 86.4% of VUS had similar/identical functional consequence to nearby pathogenic variants, which suggested their likely pathogenicity and potential molecular pathology. The distinct proteotypic consequence of CNGA3 mutants shown in our analysis strongly supported the notion that gene supplementation may be the most widely applicable therapeutic option for *CNGA3-*associated achromatopsia patients.

**Conclusion:**

Thus, proteoform-based analysis can be a valuable approach for assessing novel variants and complement clinical genomics in its utilization.

**Supplementary Information:**

The online version contains supplementary material available at 10.1186/s13023-025-03792-3.

## Background

Achromatopsia (ACHM; OMIM #216900) is a rare, congenital autosomal recessive retinal disease with cone-specific dysfunction [1], which affects one in 30,000–50,000 live births worldwide [2]. The clinical presentation of achromatopsia often includes reduced or complete loss of color perception, photophobia, poor visual acuity (< 0.2), and nystagmus [3]. These phenotypes are typically either congenital or present in infancy. To date, pathogenic variants in six genes have been associated with achromatopsia, five of which play vital roles in the cone phototransduction cascade (i.e., *CNGA3*,* CNGB3*, *GNAT2*, *PDE6C*, *PDE6H*, and ATF6) [4]. Up to 70% of achromatopsia patients carry pathogenic mutations in *CNGB3*, while another 25% of patient cases have pathogenic *CNGA3* variants [1, 5, 6]. Notably, CNGA3 and CNGB3 function together by forming a heterotetramer cyclic nucleotide-gated (CNG) channel resulting in up to 95% of achromatopsia patients carrying pathogenic defects in the CNGA3/CNGB3 channel complex (cone CNG channel) [6, 7]. 

The cone CNG channel is made up of CNGA3 (α subunit) and CNGB3 (β subunit) in a 3:1 (α:β) stoichiometry [8, 9]. The CNG channel pumps cations into the cell resulting in a release of glutamate. When the channel is closed, the cone cells become hyperpolarized and glutamate release diminishes, which prompts the phototransduction cascade to continue through bipolar cells [9, 10], [11] Pathogenic variants in *CNGA3* and *CNGB3* typically result in loss of function, which suggests that CNG channel function is crucial for the phototransduction of color vision [12]. Interestingly, some of the pathogenic variants in *CNGA3* display either cone dystrophy alone (CD; progressive cone degeneration) or both ACHM and CD. This suggests that CNGA3 is important for cone viability [12]. Therefore, understanding the molecular pathology of mutations in cone CNG channels in ACHM patients is desirable for the development of therapeutic strategies like *CNGA3-* and *CNGB3-*targeting gene therapies. Trials concerning *CNGA3*-associated achromatopsia gene therapy are ongoing [13]. 

As of January 2024, there were 199 *CNGA3* variants with an association to achromatopsia in ClinVar with at least one peer-reviewed research article discussing the variant [1]. Of the 199 variants, 49 were either nonsense or frameshift mutations. The remaining 150 variants were missense mutations, whereof 47 were classified as pathogenic and 103 were variants of unknown significance (VUS). This suggests that the pathogenicity of more than half of the *CNGA3* patient variants, mainly missense, could not be determined by clinical genomics alone and additional patient variant analysis could be beneficial [14]. 

In this study, we present an achromatopsia patient with a novel homozygous variant in the *CNGA3* gene. We established a three-dimensional protein structure-based proteoform analysis of the CNGA3/CNGB3 complex to better understand CNG channel-dependent achromatopsia pathology and determined the pathogenicity and potential disease pathology of our patient’s mutation. We also performed clinical functional tests (Optical Coherence Tomography (OCT), fundus imaging and fundus autofluorescence imaging) of our index patient. Our proteoform analysis was further extended to study the structural and functional consequences of known pathogenic variants. Based on our findings from the proteoform analysis of pathogenic variants, we suggest potential molecular defects of ACHM patient VUS.

## Methods

### Study approval

The study adhered to the Tenets of the Declaration of Helsinki and ethics approval was granted by Stanford University Institutional Review board. Informed consent was obtained from the patient.

### Clinical imaging

Spectral-domain optical coherence tomography (SD-OCT) and short-wavelength fundus autofluorescence (FAF) images were taken (Spectralis HRA + OCT device; Heidelberg Engineering, Heidelberg, Germany).

### Genetic sequencing and analysis

The patient’s genetic sequence analysis was performed using the Blueprint Genetics My Retina Tracker panel. Variants were identified using ClinVar and classified as “demonstrated links to ocular clinical phenotypes” if there were publications discussing the variant. If publications did not previously discuss a variant, it was classified as novel.

### Structural analysis of CNGA3 variants

The cryogenic electron microscopy (CryoEM) structure of the CNGA3/CNGB3 channel complex (PDB ID: 7RHS) was used for structural analysis. The experimentally determined CNGA3/CNGB3 channel complex was composed of three CNGA3 molecules and one CNGB3 molecule. The figure was generated using Pymol (The PyMOL Molecular Graphics System, Version 2.0 Schrödinger, LLC).

### Location calculation

A plot of the distance from each residue to the central axis and the center of the membrane was curated based on the coordinates of each residue’s ⍺-carbon from the PDB file. The central axis was defined as the line running through both the central Na^+^ ion and the Center of Mass calculated in Pymol. As the protein was symmetrically arranged around the channel, the Center of Mass function in pymol was suitable as a second point. The center of the membrane was defined as a plane perpendicular to the channel axis in the middle of the transmembrane domains.

### Consurf analysis

The conservation of CNGA3 across 58 primate sequences from UniProtKB was calculated using the Multi-Sequence Alignment (MSA) tool on Consurf [15] along with the PDB file for CNGA3. A conservation score was determined for each residue.

## Results

### Clinical findings

**Fig. 1:**
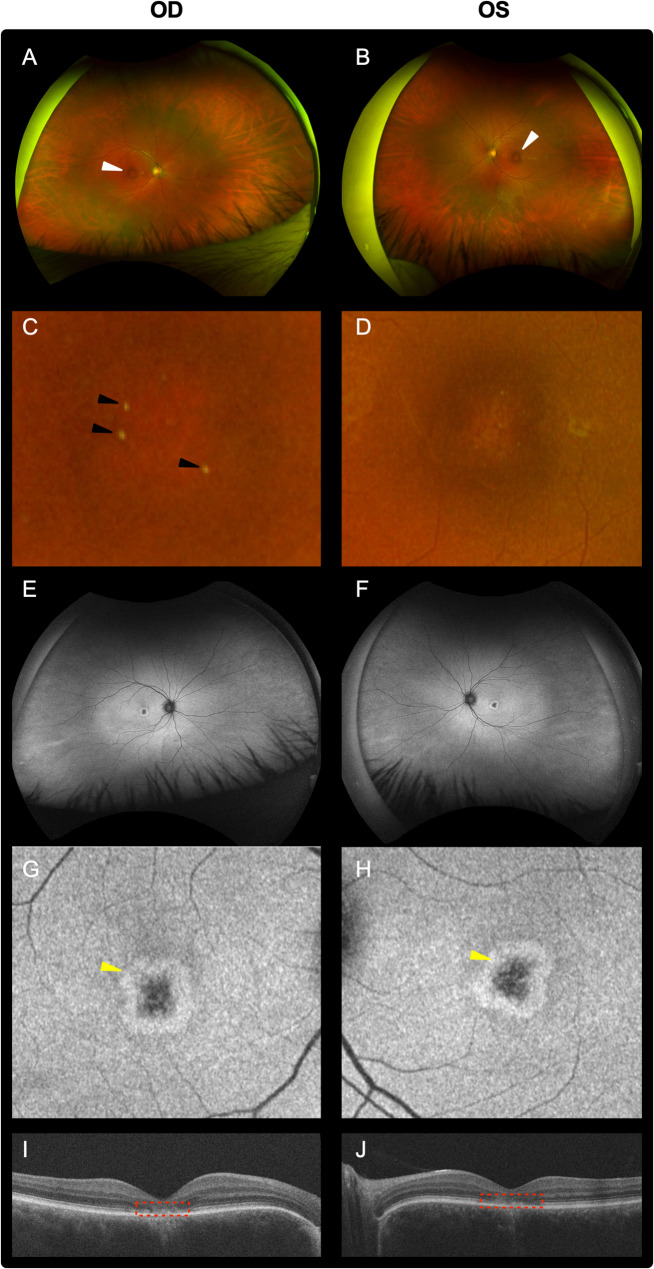
Clinical imaging. Fundus imaging (**A-D**) and auto-fluorescent imaging (**E-H**) showed normal discs and vessels. A mild hyperpigmentation around the fovea is seen in both eyes (panel A; white arrows) and a few drusen are apparent in the right eye (black arrows). Yellow arrows show the auto-fluorescent ring. OCT (**I-J**) shows thinning at the fovea with photoreceptor loss (red arrow). Cystoid macular edema, an epiretinal membrane, or subretinal fluid were not observed

A 34-year-old male without color perception had experienced photophobia and nystagmus since infancy. Co-morbidities included lumbar disc herniation, vitamin B12 deficiency, disorder of the eustachian tube, depression, and generalized anxiety disorder. No one in his extended family possessed a history of retinal disease. On exam, his visual acuity was 20/150 for the right eye (OD) and 20/150 for the left eye (OS). Intraocular pressure was recorded at 14 mmHg OD and 16 mmHg OS. Slit lamp examination revealed a normal anterior segment in both eyes. Vitreous syneresis was apparent OU. Fundus examination revealed a dark ring around the fovea in both eyes (Fig. 1A-D; white arrow) as well as a few drusen in the right eye (Fig. 1A and C; black arrows). The disc and the vessels as well as the periphery appeared normal (Fig. 1E-H). Optical coherence tomography showed thinning of the fovea with photoreceptor loss in both eyes (Fig. 1I-J; red box). No cystoid macular edema, epiretinal membrane, or subretinal fluid was observed. The findings were consistent with achromatopsia.

### Genetic analysis

Genetic testing revealed a novel homozygous *CNGA3* c.587 A > T, p.Gln196Leu variant. This variant was absent in disease-related genomic variation databases such as ClinVar, HGMD as well as gnomAD, a large reference population database (*n* > 120,000 exomes and > 15,000 genomes). This suggested that the variant was very rare in the general population. There were no variants in other genes associated with achromatopsia (Supplemental Text S1). The patient’s parents declined genetic testing, but none of the patient’s family members possessed ocular symptoms or diagnoses related to achromatopsia (Figure S1). In silico genetic pathogenicity analysis tools resulted in inconsistent predictions (Polyphen: benign; SIFT: deleterious; and MutTaster: disease-causing), yet the affected amino acid was highly conserved in mammals as well as in evolutionarily more distant species, which suggested that this position may not tolerate variation (Figure S2).

A missense variant affecting the same codon *CNGA3* c.587 A > G, p.Gln196Arg, was previously reported in an inherited retinal dystrophy patient together with a *CNGA3* c.872_873del, p.Thr291Argfs*77 frameshift variant in a compound heterozygous manner [16]. However, there was insufficient information to declare the variant’s pathogenicity through clinical genetics analysis. Thus, the variant remained as a VUS. At the amino acid level, there was a moderate physicochemical difference between Gln and Leu (Grantham distance: 113 [0-215]; polar to hydrophobic amino acid mutation), and this mutation was possibly damaging to CNGA3 protein structure-function.

### Three-dimensional proteoform analysis

**Fig. 2:**
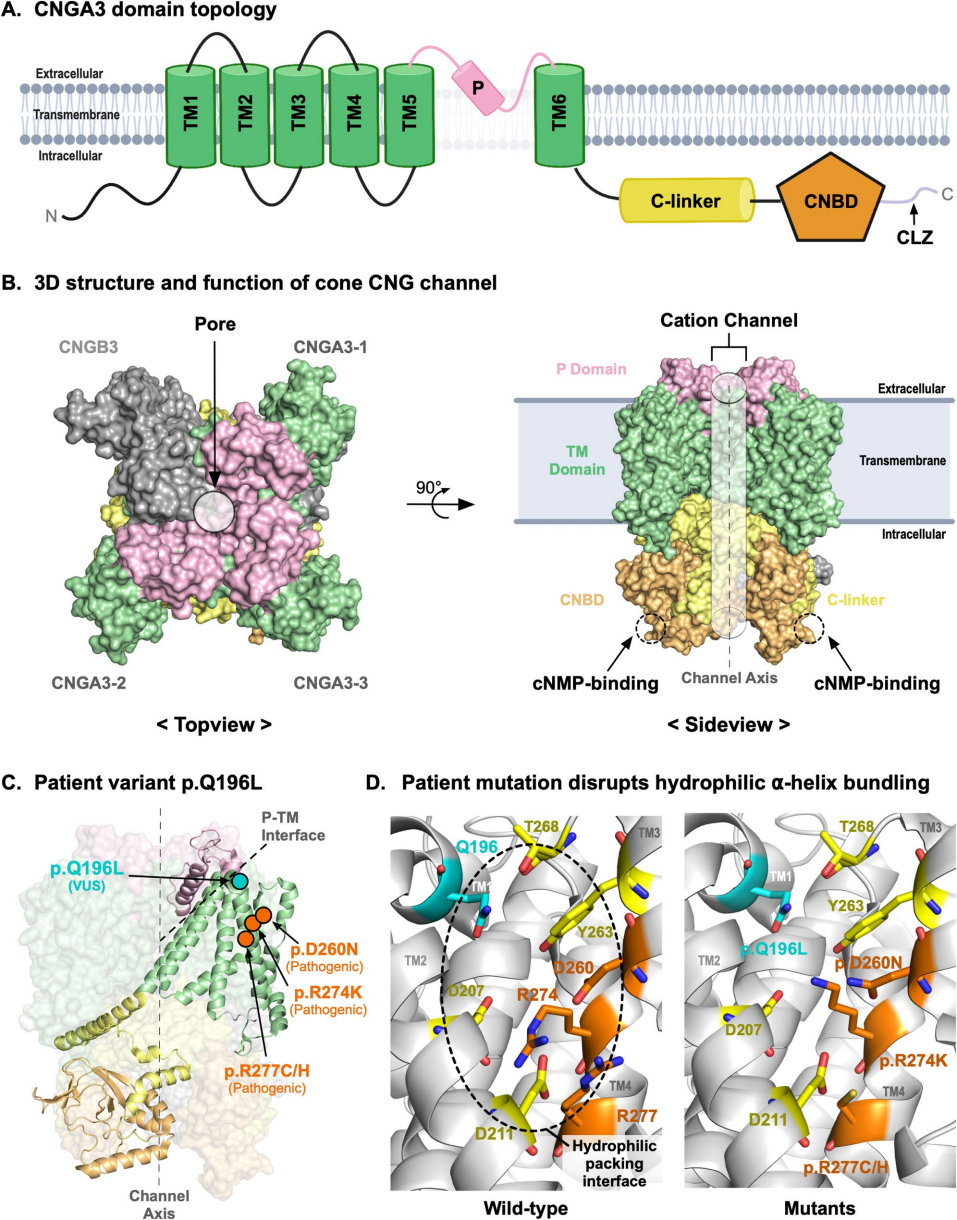
CNGA3 mutation disrupts packing in cone CNG channel formation. (**A**) CNGA3 is a multi-domain protein consisting of six channel-forming transmembrane domains (TM1-6), a pore-gating region (P), a C-linker domain, a cyclic nucleotide-binding domain (CNBD), and a CLZ (C-terminal Leucine Zipper). (**B**) The CryoEM structure of the cone CNG channel (PDBID: 7RHS) shows a heterotetramer protein complex consisting of a CNGB3 subunit and three CNGA3 subunits. The cation channel formed by the TM domains and P domain is shown as a grey cylinder and the cyclic nucleotide binding site at the CNBD domain is shown in a dotted circle. The channel allows cations to flow into the cell upon cNMP binding to CNBD. (**C**) Our patient mutation p.Q196L is located at the interface between the pore-gating region and the transmembrane domains. The previously reported pathogenic variant (p.Q196R) is also at the same location. Other pathogenic variants (p. D260N, p.R274K, and p.R277C/H) that are at the TM domain packing site are highlighted in orange. (**D**) The Q196 residue helps tight packing of alpha-helices of TM domains through hydrogen bond interactions with T268 residue. Our patient mutation causes loss of this interaction. Pathogenic variants p. D260N, p.R274K, and p.R277C/H display identical molecular consequences as p.Q196R at the same alpha helix packing region

CNGA3 is a multi-domain transmembrane protein that consists of six transmembrane helices (TM1-6; also known as transmembrane segments), a pore-gating region [also known as central pore (P)], a C-linker, a cyclic nucleotide-binding domain (CNBD), and a cytosolic C-terminal leucine zipper (CLZ) (Fig. 2A). The three-dimensional (3D) protein structure of the CNGA3/CNGB3 channel complex, determined by cryogenic electron microscopy (CryoEM; PDBID: 7RHS), showed that three CNGA3 molecules and one CNGB3 molecule formed a cone-specific heterotetrameric CNG channel [9]. The CLZ domain facilitated the formation of the CNG channel complex [8], while other domains served distinct roles for the channel function. The transmembrane domains and extracellular pore-gating region formed a channel for intracellular cation transport. The CNBD underwent a conformational change when cyclic nucleotides (e.g., cGMP) bind [9], and the C-linker domain then conveyed this conformational change to the channel forming domains (i.e., TMs and P) to be in a closed channel conformation (Fig. 2B) [11, 17–19]. Thus, the structural and conformational orchestration of these domains in response to cyclic nucleotide binding was a critical molecular regulatory mechanism for this channel. In addition, most of the known disease-causing mutations were missense and the protein sequences of CNGA3 among primates (species that have highly similar color vision as human) were highly conserved (Figure S2). This implied that the CNGA3 structure-function may not tolerate small variations in amino acids [20, 21]. 

We mapped our patient’s variant [p.Gln196Leu (p.Q196L)] to the 3D structure of the cone CNG channel complex. This mutation was located near the interface between the TMs and P (Fig. 2C). More specifically, it was at a position between TM1 and TM2 contributing to a certain transmembrane conformation by tightly bundling multiple alpha-helices together. The wild-type Gln196 (glutamine at residue 196) and its surrounding residues such as Asp207, Asp211, Asp260, Tyr263, Thr268, Arg274, and Arg277 formed a hydrophilic ⍺-helical packing interface, while our patient’s leucine mutation (p.Gln196Leu) disrupted the interaction which may reduce conformational stability (ΔΔG = -0.201 kcal/mol) [22] (Fig. 2D). Notably, there was a pathogenic variant p.Gln196Arg at the same residue that also reduced conformational stability (ΔΔG = -0.989 kcal/mol). This strongly suggested that the Gln196 played a critical pathophysiological role. Additionally, we identified pathogenic variants (i.e., p.Asp260Asn, p.Arg274Lys, and p.Arg277Cys/His) [14, 23] that were closely located to our patient variant in a 3D structure, even though they were distantly located from our patient’s variant in the one-dimensional protein coding sequence. These variants were found at the alpha-helix bundling region affecting hydrophilic packing interactions and they also reduce conformational stability. Taken together, these findings strongly suggested that mutations at the ⍺-helix bundling region were one of the key molecular pathologies of ACHM causing disruption in the channel conformation. In addition, our 3D structure-based proteoform analysis, in conjunction with clinical genomics findings, strongly supported the molecular basis that our patient variant was pathogenic.

### Proteoform analysis of pathogenic missense variants revealed six variant groups with different molecular pathology

We expanded our structure-function proteoform analysis to examine 52 pathogenic *CNGA3* variants (47 missense, 3 nonsense and 2 frameshifts) to gain further insight into *CNGA3*-associated molecular pathology and attempted to redefine pathogenicity of 147 VUS (103 missense, 21 nonsense and 23 frameshift) curated from ClinVar and existing literature.

**Fig. 3:**
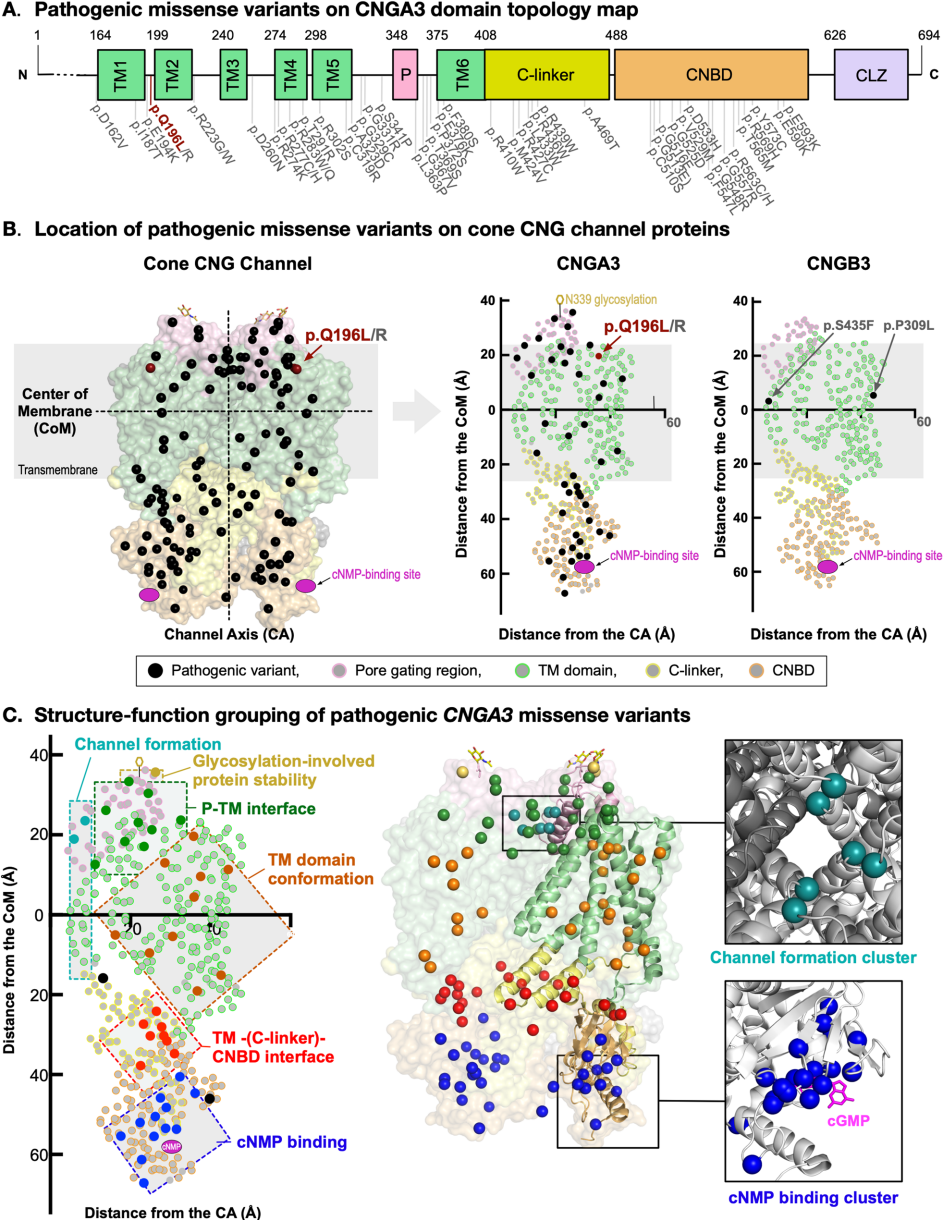
Pathogenic CNGA3 variants group by structure-function in three dimensions. (**A**) Pathogenic missense variants are distributed across *CNGA3* without apparent clustering. (**B**) Three-dimensional CNG channel proteoform model demonstrating established pathogenic missense variants on 3D-structure as well as mapped by distance to the central channel axis (CA) and distance to the center of the membrane (CoM) for both CNGA3 and CNGB3. Both subunits show similar patterns of residue distribution. (**C**) This plot revealed six distinct groups of variants based on anatomical location and molecular consequence. Variants were either located right by the channel (light blue), in the glycosylation-pore gating region which protects against degradation (yellow), at the P-TM interface (green), in the wing regions supporting proper TM domain conformation (orange), in cNMP regulation sites (red) or at the cNMP binding site (dark blue)

To identify any mutational hotspots associated with achromatopsia, 47 pathogenic missense variants were mapped on the linear CNGA3 sequence (Fig. 3A), and no clear mutational clusters/hotspots were observed. Then, we mapped these pathogenic variants on a three-dimensional cone CNG channel structure to analyze them in the context of structure-function associations (Fig. 3B). Since the CNG channel was a heterotrimer transmembrane complex forming a cation channel in the middle, we plotted each protein residue by its relative distance from the channel axis and the center of the membrane (CoM). These 2D plots containing 3D information demonstrated that CNGA3 and CNGB3 have very similar protein structures in relation to the channel. From our analysis, the pathogenic patient variants can be separated into 6 groups based on their structure-function pathology (Fig. 3C). Variants found at the central channel protrude into the channel or disrupt the channel shape which may prevent cations from flowing through the channel (Table S2; light blue). Glycosylation at N339 residue was critical for protein stability and undertaking proper cation gating (Fig. 3B) [24]. N-linked glycosylation occurred at an amino acid sequence motif defined by NX_1_(S/T)X_2_ [24, 25], and one of the variants (p.S341P [14]) introduced a mutation at S341, which was required for N339-glycosylation (Table S2; yellow). Nine of the pathogenic variants were found at the interface between the P and TM domains, where mutations either disrupted ⍺-helix formation or led to improper folding and conformational crosstalk between the P and TM domains (Table S2; green). One-fourth of the pathogenic missense variants clustered in the TM domain conformation cluster, where proper bundling of alpha helices was vital for the functional channel conformation (Table S2; orange). All these variants disrupted key molecular interactions that contributed to the conventional folding and packing of transmembrane domains. This occurred through either disruption of the hydrophobic core, charge-charge interaction, or charge-dipole interactions. A cluster of pathogenic variants (8 variants) was found on the interface among three domains (i.e., TM domains, C-linker, and CNBD) where the channel open state was reliant upon cyclic nucleotide monophosphate (cNMP)-binding to the CNBD. This cNMP-binding was then structurally relayed to the transmembrane domains. These variants compromised the conformational signaling response through the loss of intramolecular interaction. Additionally, some variants disturbed intermolecular interactions, preventing the formation of CNG channel tetramer (Table S2; red). Each CNG channel subunit had a cNMP binding pocket in which amino acids either directly bind to cNMP or were important for the conformation of the binding pocket. Since the binding pocket was closely located to other neighboring CNG channel subunits, this region was important for tetramer interaction. Thirteen pathogenic variants clustered around the cNMP binding pocket and disturbed its function by either altering residues that directly interact with cNMP or changing the formation of the cNMP-binding pocket. These variants ultimately resulted in channel defects (Table S2; blue).

### Proteoform analysis of missense VUS provided mechanistic insight into potential pathogenicity

**Fig. 4:**
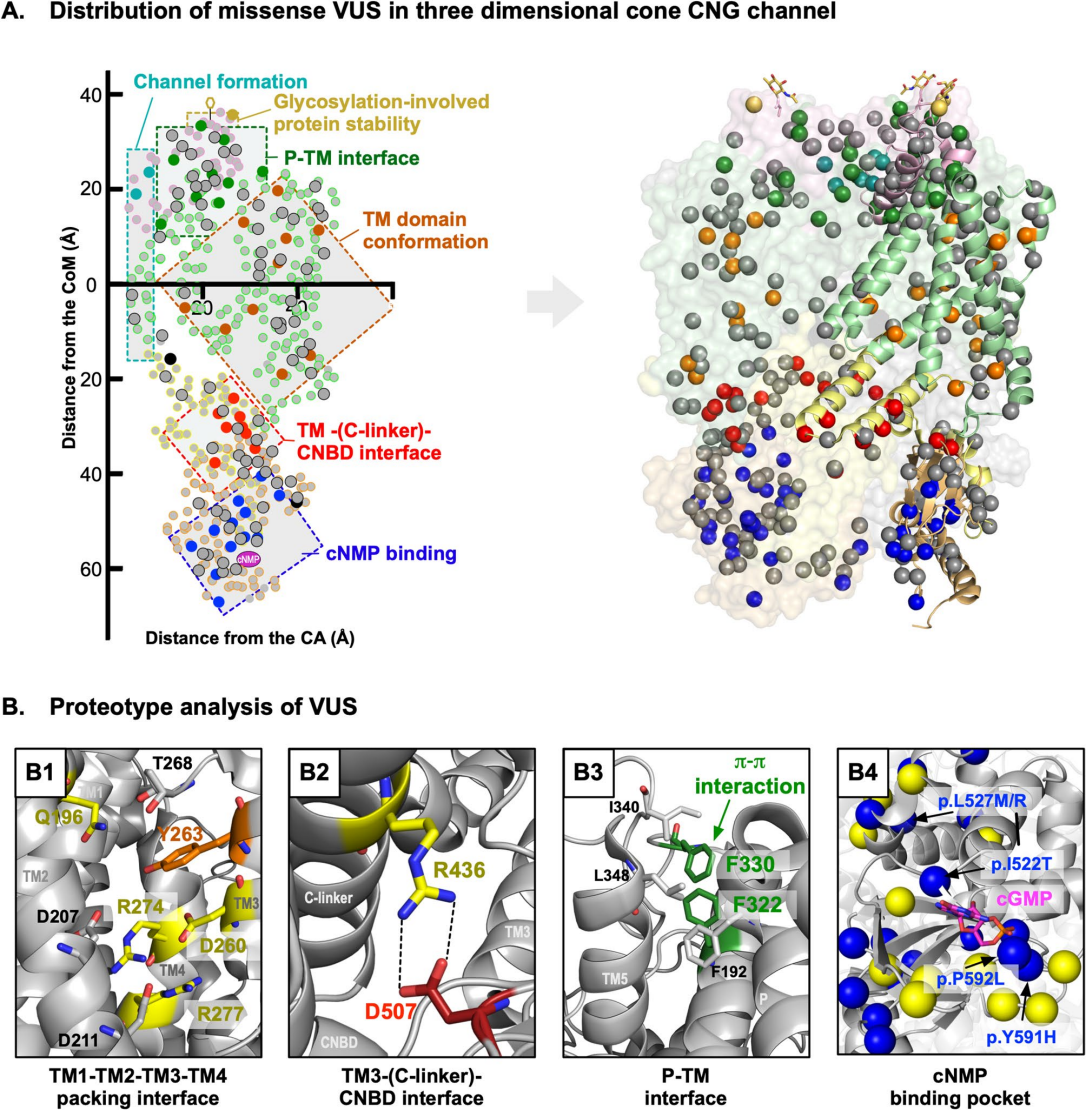
Structure-function analysis refines pathogenicity of missense VUS. (**A**) 103 missense VUS identified in literature and ClinVar were plotted on the three-dimensional protein structure as well as the two-dimensional model. Most missense VUS were found within the six groups arranged from the established pathogenic variants. (**B**) VUS were analyzed in comparison to established pathogenic variants within the same structure-function group that were either found at the same residue, disturbed the same alpha helix bundling or tight packing interfaces (**B1**), caused disruption to the same interaction (**B2**) or other VUS (**B3**) or caused structural changes to important regions like the nNMP binding site (**B4**)

Based on our findings from the pathogenic variants, 103 missense VUS were analyzed via the same 3D structure-based approach. These *CNGA3* VUS could not be declared pathogenic by clinical genomics, because they did not have conclusive genetic information. Each variant was plotted along with the pathogenic variants (Fig. 4A), and the structural consequence from each mutation was analyzed and compared to known pathogenic variants, respectively.

Fourteen (~ 13.6%) missense VUS occurred at the same residue as known pathogenic variants, even though they were not genetically identical. However, at the proteoform level, these VUS had a similar or identical molecular consequence as corresponding pathogenic variants, which strongly suggested that they were disease-causing (Table S3). For the other 89 variants without any pathogenic variants at the same residue, each VUS was analyzed in comparison with neighboring pathogenic variants and VUS in a 3D context (Fig. 4A). The majority of these VUS (75 variants, ~ 84.3%) were not only structurally close to the pathogenic variants in the corresponding structure-function groups, but also had very similar potential molecular defects to the pathogenic variants (Table S4). For example, the *CNGA3* p.Tyr263Asp [26] VUS was found in the TM conformation interface where our patient variant p.Gln196Leu and pathogenic variant p.Arg274Lys [23] were found. Along with Gln196 and Arg274, Tyr263 was one of the key residues contributing to the tight hydrophilic packing of TM1, TM2, TM3, and TM4 α-helices. These variants all disrupted the proper α-helical bundling by substituting large, polar residues with residues that were shorter or hydrophobic in their chemical properties (Fig. 4B1). Several other VUS had a direct association with pathogenic variants which can only be identified in a 3D structural context. Arg436 and Asp507 [27] made direct charge-charge interaction at the TM3-(C-linker)-CNBD interface, where both pathogenic variant p.Arg436Trp [14] and VUS p.Asp507Gly^5^ resulted in loss of this interaction (Fig. 4B2). There were also cases where two VUS hinted towards pathogenicity of each other in a 3D context. π-π stacking interaction between Phe322 and Phe330 help hydrophobic core interactions at the P-TM interface. There were two VUS, p.Phe322Ser and p.Phe330Ser [23], substituting each phenylalanine for serine and thus disrupting the interaction identically. Since both variants were identified in achromatopsia patients and caused identical molecular defects, it was highly likely that they were disease-causing (Fig. 4B3). Notably, there was a large group of VUS (25 variants including p.Ile522Thr [14], p.Leu527Arg/Met [27, 28], p.Tyr591His [1], and p.Pro592Leu [1]) found at the CNBD domain near the cNMP binding site (Fig. 4A and B**4**). The 13 pathogenic variants in this region served as molecular precedents which suggested that the VUS in this region also caused either loss of direct interaction with cNMP molecules or disturbed the conformation of the cNMP binding pocket. Such losses of interaction and disturbances can prevent channel activation by prohibiting the accurate binding of cNMPs.

### Proteoform analysis of nonsense and frameshift variants suggested that gene supplementation/augmentation was a desired therapeutic strategy for CNGA3-related achromatopsia

At the transcription regulation level, most of the nonsense and frameshift mutations occurring before exon 8 (*n* = 9) were likely to induce mRNA decay resulting in no translation of protein. For the remaining nonsense and frameshift variants, we mapped the pathogenic variants on the CNGA3 domain topology map (Figure S3). In case of partial protein translation, mutations resulting in defects before or within TM6 would prevent proper CNGA3 folding. Premature termination variants after the transmembrane domains would also result in loss of CNG-channel function since the following C-linker and CNBD domains were the cyclic nucleotide-dependent structural regulators of channel function. These proteotypic molecular pathologies applied to every pathogenic *CNGA3* nonsense and frameshift variant. Next, we examined 33 VUS (23 nonsense and 10 frameshift) identified in patients (Figure S3 and Table S1) and found 94% (31 variants) introduced protein truncation earlier than the CNBD domain (23 were found earlier than TM6; 11 were at C-linker; and 8 were at CNBD; Figure S3). These VUS were expected to exhibit proteotypic molecular consequences identical to pathogenic variants, which strongly suggested that they were disease-causing. Since achromatopsia was an autosomal recessive disease, one functioning variant was sufficient to regain color vision. With the heterogeneity of the many CNGA3 variants, gene supplementation/augmentation may be the most desirable strategy for gene therapy approaches.

In summary, proteoform analysis of patient genetic variants can complement clinical genomics in pathogenicity determination by revealing groups of variants with similar structure-function consequences and providing further insight into the potential molecular pathology of individual patient variants.

## Discussion

We reported the phenotype in a patient with *CNGA3*-mediated achromatopsia, a novel patient variant (c. 587 A > T, p.Gln196Leu), and three-dimensional protein structure-based structure-function analysis of 52 pathogenic *CNGA3* variants and more than 100 variants of unknown significance curated from literature and ClinVar. CNGA3 and CNGB3 functioned together by forming a heterotetramer cyclic nucleotide-gated (CNG) channel resulting in up to 95% of achromatopsia patients having pathogenic defects in the CNGA3/CNGB3 channel complex (cone CNG channel) [6, 7]. It is important to note that while proximity to pathogenic variants may be important, a VUS as close as a few residues upstream or downstream of a pathogenic variant could have a quite different location and function when viewed in three-dimensional space. By mapping established pathogenic variants on the CryoEM structure of cone CNG-channel, we identified 6 distinctive variant groups each with unique structure-function consequences which were not evident from a two-dimensional protein sequence. And while not as straightforward as statistical genetics, this can be translated into clinical practice by including structural biology models and structural biology expertise to bolster likely pathogenicity. This analysis provided a valuable supplement to traditional clinical genomics and revealed that the mutation likely resulted in the formation of a non-functioning cone CNG channel. Clinical examination of the affected patient and chart review confirmed the diagnosis of achromatopsia, which was consistent with the structure-function analysis. As in many cases during regular clinical practice, family genetic testing was not available to confirm VUS segregation. Nonetheless, protein structure analysis in such cases may be particularly helpful in determining the likelihood of pathogenicity*.*

The proteoform analysis supported clinical genomics in genetic counseling and assisted therapeutic development for achromatopsia patients. Since *CNGA3* variants caused cone CNG channel function loss by disturbing the very conserved protein structure and conformation, different therapeutic approaches can be considered. One possible approach is the development of drug therapies, which could enhance the function of the cone CNG channel. For example, variants at the cNMP-binding site may benefit from synthetic cNMP-analogues that are tailored to overcome structural conformational defects of the binding pocket [29, 30]. However, this approach may not broadly restore CNG channel function in all variants, because each variant affects channel function through distinct molecular mechanisms as revealed in our proteoform analysis. Instead, gene therapy may be useful in restoring a functioning cone CNG channel. Since CNGA3-associated achromatopsia is inherited in autosomal recessive manner, one functioning allele should be sufficient to restore color vision. This supports that gene therapy by gene supplementation may be the most widely applicable approach for this autosomal recessive disease [12]. Clinical studies of *CNGA3* gene supplementation have been conducted with promising results for achromatopsia [12, 13, 31, 32]. 

Although our structure-function model can provide insight into clinical genomics, it does have limits. Notably, there were 2 VUS found at the CLZ domain which facilitates the formation of the tetrameric CNG channel complex. The variants may interfere with the formation of the CNG channel, but some amino acids in the CLZ domain were not included in the CryoEM model and were therefore not analyzed further. Although, studies suggest the CLZ domain is vital to tetramer formation due to a very compact hydrophobic conformation and introduction of polar residues at sites with hydrophobic interaction will likely result in disease [8]. For these variants, along with variants potentially interruption glycosylation patterns, experimental testing is necessary to conclusively determine the pathogenicity. Furthermore, our analysis is limited to variant information found in the literature and ClinVar which does not include extensive gene sequencing information, but only specific DNA variants identified in each patient (Table S3).

Our approach is specifically tailored to compliment clinical genomics in determining pathogenicity in CNGA3-associated achromatopsia, but future perspectives include extrapolation to non-Mendelian disorders with coding variants and prominent effects on a functional protein level. Determining benign variants can be supported if there is sufficient data with VUS frequency at a population level and pedigree studies– something not always available in ClinVar - where such variants could be mapped into three-dimensional protein structures. Population based variant incidences and pedigree studies were not part of the current study, but computationally more intensive structure-function analysis is possible in future studies*.*

## Conclusion

Based on our findings, 3D proteoform-based structure-function analysis of *CNGA3* variants in achromatopsia is a useful approach in pathogenicity determination, and reveals strong likelihood of pathogenicity of both our patient variant *CNGA3* c.587 A > T, p.Gln196Leu as well as in 86.4% of *CNGA3* VUS reported in ClinVar and the literature.

## Electronic Supplementary Material

Below is the link to the electronic supplementary material.

Supplementary Material 1

## Data Availability

All data generated or analyzed during this study are included in this published article and its supplementary information files.

## References

[1] Solaki M, Baumann B, Reuter P, Andreasson S, Audo I, Ayuso C, et al. Comprehensive variant spectrum of the CNGA3 gene in patients affected by achromatopsia. Hum Mutat. 2022;43(7):832–58. 35332618 10.1002/humu.24371

[2] Brunetti-Pierri R, Karali M, Melillo P, Di Iorio V, De Benedictis A, Iaccarino G et al. Clinical and molecular characterization of achromatopsia patients: A longitudinal study. Int J Mol Sci. 2021;22(4). 10.3390/ijms22041681 PMC7914547 33562422

[3] Pascual-Camps I, Barranco-Gonzalez H, Aviñó-Martínez J, Silva E, Harto-Castaño M. Diagnosis and treatment options for achromatopsia: A review of the literature. J Pediatr Ophthalmol Strabismus. 2018;55(2):85–92. 29257187 10.3928/01913913-20171117-01

[4] Zobor D, Zobor G, Kohl S. Achromatopsia: on the doorstep of a possible therapy. Ophthalmic Res. 2015;54(2):103–8. 26304472 10.1159/000435957

[5] Hirji N, Aboshiha J, Georgiou M, Bainbridge J, Michaelides M. Achromatopsia: clinical features, molecular genetics, animal models and therapeutic options. Ophthalmic Genet. 2018;39(2):149–57. 29303385 10.1080/13816810.2017.1418389

[6] Remmer MH, Rastogi N, Ranka MP, Ceisler EJ. Achromatopsia: a review. Curr Opin Ophthalmol. 2015;26(5):333–40. 26196097 10.1097/ICU.0000000000000189

[7] Michalakis S, Gerhardt M, Rudolph G, Priglinger S, Priglinger C. Achromatopsia: genetics and gene therapy. Mol Diagn Ther. 2022;26(1):51–9. 34860352 10.1007/s40291-021-00565-z PMC8766373

[8] Zhong H, Molday LL, Molday RS, Yau KW. The heteromeric Cyclic nucleotide-gated channel adopts a 3A:1B stoichiometry. Nature. 2002;420(6912):193–8. 12432397 10.1038/nature01201 PMC2877395

[9] Zheng X, Hu Z, Li H, Yang J. Structure of the human cone photoreceptor Cyclic nucleotide-gated channel. Nat Struct Mol Biol. 2022;29(1):40–6. 34969976 10.1038/s41594-021-00699-y PMC8776609

[10] Ding XQ, Matveev A, Singh A, Komori N, Matsumoto H. Biochemical characterization of cone Cyclic nucleotide-gated (CNG) channel using the infrared fluorescence detection system. Adv Exp Med Biol. 2012;723:769–75. 22183405 10.1007/978-1-4614-0631-0_98 PMC3370941

[11] Luo DG, Su CY, Yau KW. Photoreceptors: physiology. In: Squire LR, editor. Encyclopedia of neuroscience. Oxford: Academic; 2009. pp. 677–86.

[12] Hassall MM, Barnard AR, MacLaren RE. Gene therapy for color blindness. Yale J Biol Med. 2017;90(4):543–51. 29259520 PMC5733843

[13] Reichel FF, Michalakis S, Wilhelm B, Zobor D, Muehlfriedel R, Kohl S, et al. Three-year results of phase I retinal gene therapy trial for CNGA3-mutated achromatopsia: results of a Non randomised controlled trial. Br J Ophthalmol. 2022;106(11):1567–72. 10.1136/bjophthalmol-2021-319067 34006508

[14] Wissinger B, Gamer D, Jägle H, Giorda R, Marx T, Mayer S, et al. CNGA3 mutations in hereditary cone photoreceptor disorders. Am J Hum Genet. 2001;69(4):722–37. 11536077 10.1086/323613 PMC1226059

[15] Berezin C, Glaser F, Rosenberg J, Paz I, Pupko T, Fariselli P, et al. ConSeq: the identification of functionally and structurally important residues in protein sequences. Bioinformatics. 2004;20(8):1322–4. 14871869 10.1093/bioinformatics/bth070

[16] Sun W, Li S, Xiao X, Wang P, Zhang Q. Genotypes and phenotypes of genes associated with achromatopsia: A reference for clinical genetic testing. Mol Vis. 2020;26:588–602. 32913385 PMC7479066

[17] Gill JS, Georgiou M, Kalitzeos A, Moore AT, Michaelides M. Progressive cone and cone-rod dystrophies: clinical features, molecular genetics and prospects for therapy. Br J Ophthalmol. 2019;103(5):711–20. 30679166 10.1136/bjophthalmol-2018-313278 PMC6709772

[18] Napolitano LM, Bisha I, De March M, Marchesi A, Arcangeletti M, Demitri N, et al. A structural, functional, and computational analysis suggests pore flexibility as the base for the poor selectivity of CNG channels. Proc Natl Acad Sci U S A. 2015;112(27):E3619–28. 26100907 10.1073/pnas.1503334112 PMC4500290

[19] Napolitano LMR, Marchesi A, Rodriguez A, De March M, Onesti S, Laio A, et al. The permeation mechanism of organic cations through a CNG mimic channel. PLoS Comput Biol. 2018;14(8):e1006295. 30071012 10.1371/journal.pcbi.1006295 PMC6091977

[20] Johnson S, Michaelides M, Aligianis IA, Ainsworth JR, Mollon JD, Maher ER, et al. Achromatopsia caused by novel mutations in both CNGA3 and CNGB3. J Med Genet. 2004;41(2):e20. 14757870 10.1136/jmg.2003.011437 PMC1735666

[21] Chen XT, Huang H, Chen YH, Dong LJ, Li XR, Zhang XM. Achromatopsia caused by novel missense mutations in the CNGA3 gene. Int J Ophthalmol. 2015;8(5):910–5. 26558200 10.3980/j.issn.2222-3959.2015.05.10 PMC4630996

[22] Pires DE, Ascher DB, Blundell TL. mCSM: predicting the effects of mutations in proteins using graph-based signatures. Bioinformatics. 2014;30(3):335–42. 24281696 10.1093/bioinformatics/btt691 PMC3904523

[23] Li S, Huang L, Xiao X, Jia X, Guo X, Zhang Q. Identification of CNGA3 mutations in 46 families: common cause of achromatopsia and cone-rod dystrophies in Chinese patients. JAMA Ophthalmol. 2014;132(9):1076–83. 24903488 10.1001/jamaophthalmol.2014.1032

[24] Meighan SE, Meighan PC, Rich ED, Brown RL, Varnum MD. Cyclic nucleotide-gated channel subunit glycosylation regulates matrix metalloproteinase-dependent changes in channel gating. Biochemistry. 2013;52(46):8352–62. 24164424 10.1021/bi400824x PMC4657727

[25] Poon AF, Lewis FI, Pond SL, Frost SD. Evolutionary interactions between N-linked glycosylation sites in the HIV-1 envelope. PLoS Comput Biol. 2007;3(1):e11. 17238283 10.1371/journal.pcbi.0030011 PMC1779302

[26] Nishiguchi KM, Sandberg MA, Gorji N, Berson EL, Dryja TP. Cone cGMP-gated channel mutations and clinical findings in patients with achromatopsia, macular degeneration, and other hereditary cone diseases. Hum Mutat. 2005;25(3):248–58. 15712225 10.1002/humu.20142

[27] Lam K, Guo H, Wilson GA, Kohl S, Wong F. Identification of variants in CNGA3 as cause for achromatopsia by exome sequencing of a single patient. Arch Ophthalmol. 2011;129(9):1212–7. 21911670 10.1001/archophthalmol.2011.254

[28] Wang X, Wang H, Cao M, Li Z, Chen X, Patenia C, et al. Whole-exome sequencing identifies ALMS1, IQCB1, CNGA3, and MYO7A mutations in patients with leber congenital amaurosis. Hum Mutat. 2011;32(12):1450–9. 21901789 10.1002/humu.21587 PMC3943164

[29] Maronde E. Cyclic nucleotide (cNMP) analogues: past, present and future. Int J Mol Sci. 2021;22(23). 10.3390/ijms222312879 PMC8657615 34884683

[30] Brown RL, Strassmaier T, Brady JD, Karpen JW. The Pharmacology of Cyclic nucleotide-gated channels: emerging from the darkness. Curr Pharm Des. 2006;12(28):3597–613. 17073662 10.2174/138161206778522100 PMC2467446

[31] Fischer MD, Michalakis S, Wilhelm B, Zobor D, Muehlfriedel R, Kohl S, et al. Safety and vision outcomes of subretinal gene therapy targeting cone photoreceptors in achromatopsia: A nonrandomized controlled trial. JAMA Ophthalmol. 2020;138(6):643–51. 32352493 10.1001/jamaophthalmol.2020.1032 PMC7193523

[32] El Moussawi Z, Boueiri M, Al-Haddad C. Gene therapy in color vision deficiency: a review. Int Ophthalmol. 2021;41(5):1917–27. 33528822 10.1007/s10792-021-01717-0

