# Supplementary material for: Structure-function analysis of CNGA3-associated achromatopsia patient variants complements clinical genomics in pathogenicity determination
Source: Orphanet J Rare Dis. 2025 May 30;20:261. doi: 10.1186/s13023-025-03792-3 (PMC12125802; doi:10.1186/s13023-025-03792-3)
Supplement: Supplementary file 1 — Supplementary Material 1 [file 13023_2025_3792_MOESM1_ESM.pdf]

Supplementary Contents

<u>Table of Contents</u>

Supplemental Text

Text S1. Additional patient genetic information

Supplemental Figures

Figure S1. Patient's pedigree

Figure S2. CNGA3 3D-structural protein model with conservation scores

Figure S3. CNGA3 frameshift and nonsense variants mapped on domain topology

Supplemental Tables

Table S1. Summary of CNGA3 nonsense and frameshift variants

Table S2. Summary of established pathogenic CNGA3 missense variants

Table S3. Summary of missense VUS in CNGA3 with overlapping pathogenic variants

Table S4. Summary of missense VUS in CNGA3 without overlapping pathogenic variants

Supplemental References

Supplemental Text

Text S1. Additional patient genetic information

Apart from the homozygous variant in the *CNGA3* gene, the patient is also heterozygous for two other variants of unknown significance. One mutation is in the gene *KCNJ13* at locus c.479T>A, p.I160N. Pathogenic variants in *KCNJ13* have been associated with autosomal recessive Leber Congenital Amaurosis[1] (LCA; MIM #614186) and autosomal dominant Snowflake Vitreoretinal Degeneration[2] (SVD; MIM #193230). The patient's reported phenotype is inconsistent with the features of *KCNJ13*-related disease. Therefore, this variant is not expected to cause the patient's clinical presentation. The patient is also heterozygous for a variant of unknown significance in the gene *MYO7A* at locus c.4096C>T, p.L1366F. Pathogenic variants in *MYO7A* are associated with autosomal recessive Usher syndrome type 1B[3] (USH1B; MIM #276900) as well as recessive and dominant non-syndromic hearing loss[4] (DFNB2; MIM #600060, and DFNA11; MIM #601317, respectively). Our patient presents with primarily ocular phenotype rather than hearing loss. Thus, the patient's clinical presentation should not be attributed to this mutation.

Supplemental Figures

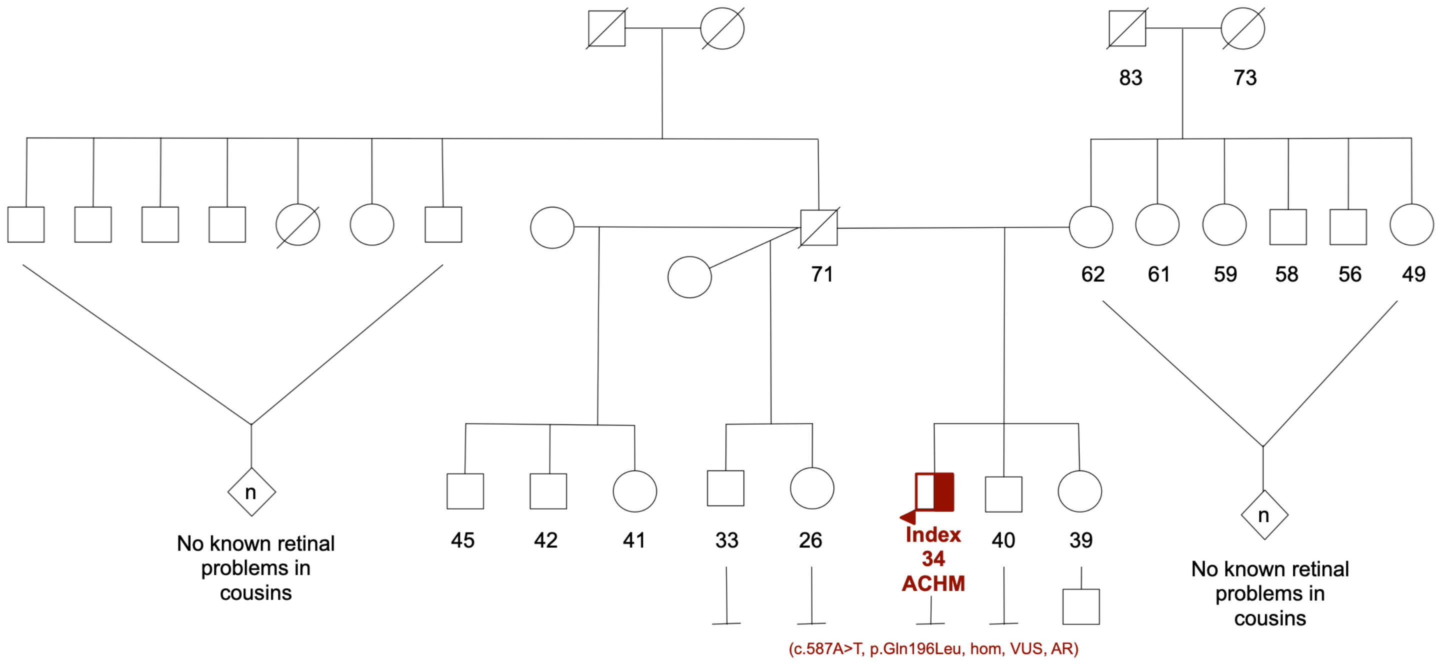

Figure S1. Patient's pedigree

Three-generational pedigree display history of retinal disease within the index patient's family. Circles represent females, and squares represent males. Symbols with a diagonal line indicate that the person is deceased. Filled symbols indicate that the person is affected with *CNGA3* c.587A>T (p.Q196L).

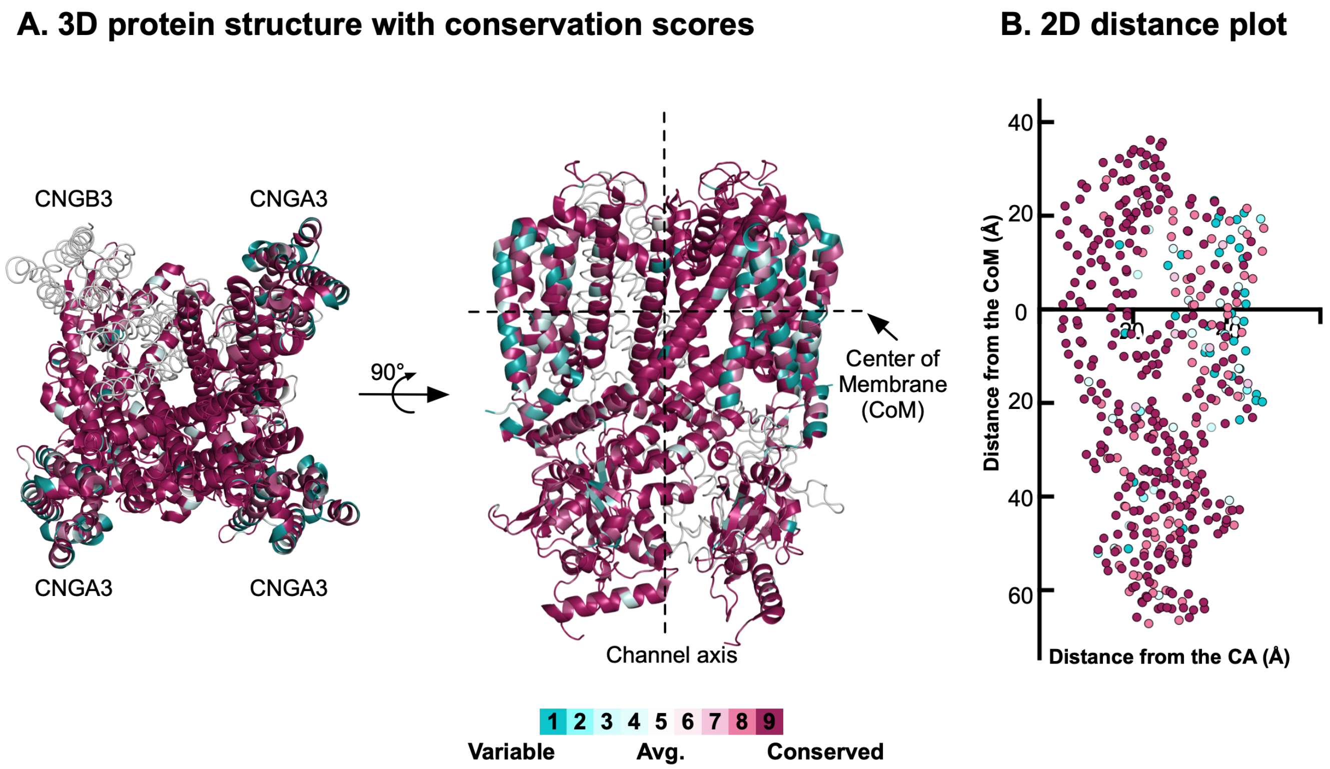

Figure S2: CNGA3 3D-structural protein model with conservation scores.

Conservation scores were determined for the CNGA3 protein using 58 primate sequences from UniprotKB and aligning them. The Multi-Sequence Alignment (MSA) was then used on ConSurf,[5] along with the cryogenic electron microscopy (CryoEM) structure of cone CNG channel complex (PDBID: 7RHS),[6] to calculate conservation scores for each residue. Each conservation score was then colored, based on the scale above, in Pymol with 1 and 9 being the most variable and conserved residues, respectively. **(A)** CryoEM structure of the cone CNG channel complex depicting conservation scores for each CNGA3 residue. **(B)** Two-dimensional plot of distance between each residue and the Center of Membrane (CoM) and Channel Axis (CA).

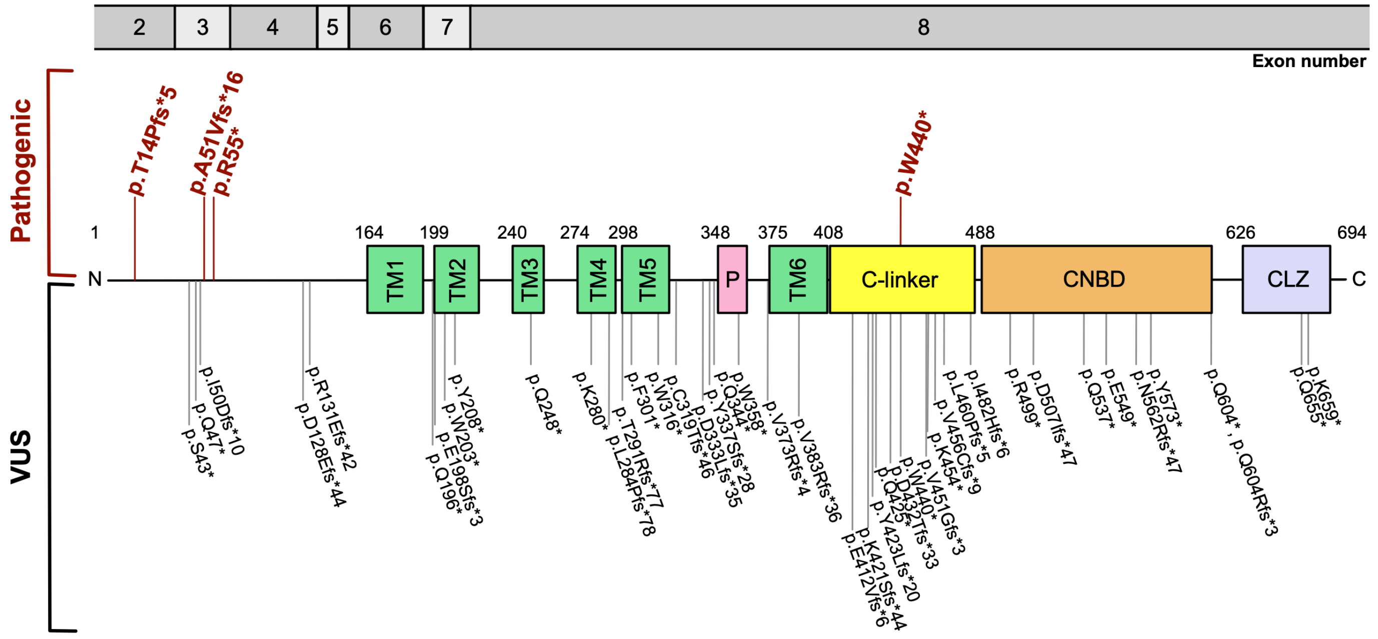

Figure S3. CNGA3 frameshift and nonsense variants mapped on domain topology

CNGA3 domain topology (TM: transmembrane domain, P: pore-gating region, CNBD: cyclic nucleotide binding domain, CLZ: C-terminal Leucine Zipper) showing frameshift and nonsense variants and their respecitve residue locations. Four pathogenic variants are labelled in burgundy. Variants of unknown significance (VUS) are shown in black: 22 variants are found earlier than or within TM6; 11 variants are found at C-linker; 8 variants are found at CNBD domain; and 2 variants are found at CLZ.

Supplemental Tables

Table S1. Summary of *CNGA3* frameshift and nonsense variants

Abbreviations: N-Term. (N-terminal region); TM (transmembrane domain); P (pore-gating region); CNBD (cyclic nucleotide binding domain); CLZ (C-terminal Leucine Zipper); cNMP (cyclic nucleotide monophosphate).

Genotype	Proteotype	Location	Proteotypic molecular pathology	Reference
**Pathogenic variants**				
c.40del	p.T14Pfs*5	N-Term.	Loss of function due to protein misfold	[7]
c.130_151dup	p.A51Vfs*16	N-Term.	Loss of function due to protein misfold	[8]
c.162_163insT	p.R55*	N-Term.	Loss of function due to protein misfold	[9]
c.1319G>A	p.W440*	C-linker	Loss of function due to protein misfold	[9]
c.1320G>A	p.W440*	C-linker	Loss of function due to protein misfold	[6]
**Variants of Unknown Significance (VUS)**				
c.139C>T	p.Q47*	N-Term.	Loss of function due to protein misfold	[26]
c.147dup	p.I50Dfs*10	N-Term.	Loss of function due to protein misfold	[6]
c.384_387del	p.D128Efs*44	N-Term.	Loss of function due to protein misfold	[5]
c.387del	p.R131Efs*42	N-Term.	Loss of function due to protein misfold	[5]
c.586C>T	p.Q196*	TM1 - TM2	Loss of function due to protein misfold	[20]
c.591del	p.E198Sfs*3	TM1 - TM2	Loss of function due to protein misfold	[5]
c.608G>A	p.W203*	TM2	Loss of function due to protein misfold	[27]
c.609G>A	p.W203*	TM2	Loss of function due to protein misfold	[25]
c.624C>G	p.Y208*	TM2	Loss of function due to protein misfold	[28]
c.742C>T	p.Q248*	TM3	Loss of function due to protein misfold	[5]
c.838A>T	p.K280*	TM4	Loss of function due to protein misfold	[29]
c.851del	p.L284Pfs*78	TM4	Loss of function due to protein misfold	[5]
c.872_873del	p.T291Rfs*77	TM4	Loss of function due to protein misfold	[9]
c.902_903delinsAA	p.F301*	TM5	Loss of function due to protein misfold	[5]
c.947G>A	p.W316*	TM5	Loss of function due to protein misfold	[6]
c.955_963delinsACCAATGAAATGGAAAT	p.C319Tfs*46	TM5	Loss of function due to protein misfold	[5]
c.997_998del	p.D333Lfs*35	TM5 - P	Loss of function due to protein misfold	[17]
c.1004G>A	p.W335*	TM5 - P	Loss of function due to protein misfold	[10]

c.1010_1012delins CAATCCCAGTG	p.Y337Sfs*28	TM5 - P	Loss of function due to protein misfold	11
c.1030G>T	p.E344*	TM5 - P	Loss of function due to protein misfold	12
c.1074G>A	p.W358*	P	Loss of function due to protein misfold	11
c.1116dup	p.V373Rfs*4	P -TM6	Loss of function due to protein misfold	11
c.1146dup	p.V383Rfs*36	TM6	Loss of function due to protein misfold	11
c.1235_1236del	p.E412Vfs*6	C-linker	Loss of cNMP-dependent regulation	23
c.1262del	p.K421Sfs*44	C-linker	Loss of cNMP-dependent regulation	24
c.1267dup	p.Y423Lfs*20	C-linker	Loss of cNMP-dependent regulation	13
c.1273C>T	p.Q425*	C-linker	Loss of cNMP-dependent regulation	10
c.1294del	p.D432Tfs*33	C-linker	Loss of cNMP-dependent regulation	2
c.1320del	p.T440Cfs*25	C-linker	Loss of cNMP-dependent regulation	10
c.1351dup	p.V451Gfs*3	C-linker	Loss of cNMP-dependent regulation	14
c.1360A>T	p.K454*	C-linker	Loss of cNMP-dependent regulation	15
c.1366del	p.V456Cfs*9	C-linker	Loss of cNMP-dependent regulation	5
c.1379del	p.L460Pfs*5	C-linker	Loss of cNMP-dependent regulation	5
c.1443dup	p.I482Hfs*6	C-linker	Loss of cNMP-dependent regulation	16
c.1495C>T	p.R499*	CNBD	Loss of cNMP-dependent regulation	17
c.1519del	p.D507Ifs*47	CNBD	Loss of cNMP-dependent regulation	5
c.1609C>T	p.Q537*	CNBD	Loss of cNMP-dependent regulation	14
c.1645G>T	p.E549*	CNBD	Loss of cNMP-dependent regulation	10
c.1682_1683insAC GCG	p.N562Rfs*47	CNBD	Loss of cNMP-dependent regulation	10
c.1719C>G	p.Y573*	CNBD	Loss of cNMP-dependent regulation	18
c.1810C>T	p.Q604*	CNBD	Loss of cNMP-dependent regulation	19
c.1811del	p.Q604Rfs*3	CNBD	Loss of cNMP-dependent regulation	25
c.1963C>T	p.Q655*	CLZ	Potential defects in channel formation	14
c.1975A>T	p.K659*	CLZ	Potential defects in channel formation	11

Table S2. Summary of established pathogenic *CNGA3* missense variants

Abbreviations: N-Term. (N-terminal region); TM (transmembrane domain); P (pore-gating region); CNBD (cyclic nucleotide binding domain); cNMP (cyclic nucleotide monophosphate). Colors correspond to clusters on Figure 3C.

Genotype	Proteotype	Location	Structure function group	Molecular consequence of patient variant	Variant reference
c.485A>T	p.D162V	N-Term.	TM domain conformation	Introduction of new hydrophobic interactions	[14]
c.560T>C	p.I187T	TM1 - TM2	TM domain conformation	Loss of W316 interaction	[19]
c.580G>A	p.E194K	TM1 - TM2	P-TM interface	Loss of K351 interaction	[14]
c.667C>G	p.R223G	TM2	TM domain conformation	Loss of T236 interaction	[20]
c.667C>T	p.R223W	TM2	TM domain conformation	Loss of T236 interaction	[14]
c.778G>A	p.D260N	TM3	TM domain conformation	Disturbed R274 interaction	[14]
c.821G>A	p.R274K	TM4	TM domain conformation	Disturbed D207 and D260 interaction	[11]
c.829C>T	p.R277C	TM4	TM domain conformation	Loss of D211 interaction	[14]
c.830G>A	p.R277H	TM4	TM domain conformation	Disturbed D211 interaction	[14]
c.847C>T	p.R283W	TM4	TM domain conformation	Loss of E286 interaction	[21]
c.848G>A	p.R283Q	TM4	TM domain conformation	Disturbed E286 interaction	[21]
c.872C>G	p.T291R	TM4 - TM5	TM domain conformation	Physical and chemical disturbance at hydrophobic core	[21]
c.906G>T	p.R302S	TM5	TM domain conformation	Disturbed E292 interaction	[22]
c.955T>C	p.C319R	TM5	P-TM interface	Loss of key hydrophobic interaction at hydrophobic core	[23]
c.968C>A	p.A323D	TM5	P-TM interface	Physical and chemical disturbance at hydrophobic core	[24]
c.985G>T	p.G329C	TM5 - P	P-TM interface	Loss of Gly necessary for α-helix turn	[25]
c.991G>C	p.G331R	TM5 - P	P-TM interface	Loss of Gly necessary for α-helix turn	[26]
c.1021T>C	p.S341P	TM5 - P	Glycosylation-involved protein stability	Loss of N339 glycosylation	[14]
c.1088T>C	p.L363P	P	P-TM interface	Disturbed α-helix conformation	[27]
c.1100G>T	p.G367V	P - TM6	Channel formation	Protrusion into channel	[27]
c.1106C>G	p.T369S	P - TM6	Channel formation	Loss of T362 interaction	[14]
c.1114C>T	p.P372S	P - TM6	P-TM interface	Disturbed α-helix conformation	[14]

c.1126G>A	p.E376K	TM6	P-TM interface	Loss of K374 interaction	27
c.1139T>C	p.F380S	TM6	P-TM interface	Loss of key hydrophobic interaction at hydrophobic core	14
c.1228C>T	p.R410W	TM6 - C-linker	.	Loss of T293 interaction	21
c.1270A>G	p.M424V	C-linker (αA')	TM-(C-linker)-CNBD interface	Disturbed V437 interaction	28
c.1279C>T	p.R427C	C-linker (αA' - αB')	TM-(C-linker)-CNBD interface	Disturbed oligomerization with other CNG channel subunits	14
c.1298T>G	p.L433W	C-linker (αB')	TM-(C-linker)-CNBD interface	Disturbed I468 interaction	29
c.1306C>T	p.R436W	C-linker (αB')	TM-(C-linker)-CNBD interface	Loss of D507 interaction	14
c.1315C>T	p.R439W	C-linker (αB')	TM-(C-linker)-CNBD interface	Disturbed oligomerization with other CNG channel subunits	30
c.1405G>A	p.A469T	C-linker (αD')	TM-(C-linker)-CNBD interface	Physical and chemical disturbance at hydrophobic core	30
c.1529G>C	p.C510S	CNBD (β2)	cNMP binding	Disturbed cNMP binding pocket	14
c.1538G>A	p.G513E	CNBD (β2 - β3)	cNMP binding	Disturbed cNMP binding pocket	14
c.1547G>A	p.G516E	CNBD (β2 - β3)	cNMP binding	Disturbed cNMP binding pocket	14
c.1574G>A	p.G525D	CNBD (β3 - β4)	TM-(C-linker)-CNBD interface	Disturbed oligomerization with other CNG channel subunits	14
c.1585G>A	p.V529M	CNBD (β4)	cNMP binding	Steric hindrance in β-sheet	21
c.1597G>C	p.D533H	CNBD (β4 - β5)	.	Inconclusive	11
c.1641C>A	p.F547L	CNBD (β6)	cNMP binding	Disturbance of direct interaction with cNMP	21
c.1642G>A	p.G548R	CNBD (β6)	cNMP binding	Disturbance of direct interaction with cNMP	16
c.1669G>A	p.G557R	CNBD (β6 - β7)	cNMP binding	Disturbed cNMP binding pocket	21
c.1687C>T	p.R563C	CNBD (β6 - β7)	cNMP binding	Disturbed cNMP binding pocket	29
c.1688G>A	p.R563H	CNBD (β6 - β7)	cNMP binding	Disturbed cNMP binding pocket	14
c.1694C>T	p.T565M	CNBD (β6 - β7)	cNMP binding	Disturbance of direct interaction with cNMP	14
c.1706G>A	p.R569H	CNBD (β7)	cNMP binding	Disturbed cNMP binding pocket	14
c.1718A>G	p.Y573C	CNBD (β7 - β8)	TM-(C-linker)-CNBD interface	Loss of Y443 interaction	14
c.1768G>A	p.E590K	CNBD (αB)	cNMP binding	Disturbance of cNMP binding pocket / Disturbed oligomerization with other CNG channel subunits	12
c.1777G>A	p.E593K	CNBD (αC)	cNMP binding	Disturbance of direct interaction with cNMP / Disturbed oligomerization with other CNG channel subunits	14

Table S3. Summary of missense VUS in *CNGA3* with overlapping pathogenic variants

Abbreviations: N-Term. (N-terminal region); TM (transmembrane domain); P (pore-gating region); CNBD (cyclic nucleotide binding domain); cNMP (cyclic nucleotide monophosphate).

Genotype	Proteotype	Location	Structure-function group	Overlapping pathogenic variant	Molecular consequence of patient variant	Variant reference
c.778G>C	p.D260H	TM3	TM domain conformation	p.D260N	Similar consequences as pathogenic p.D260N	10
c.822G>T	p.R274S	TM4	TM domain conformation	p.R274K	Similar yet more severe consequences as pathogenic p.R274K	31
c.829C>G	p.R277G	TM4	TM domain conformation	p.R277C/H	Similar yet more severe consequences as pathogenic p.R277C/H	27
c.967G>C	p.A323P	TM5	P-TM interface	p.A323D	Physical disturbance at hydrophobic core	32
c.992G>A	p.G331E	TM5 - P	P-TM interface	p.G331R	Similar consequences as pathogenic p.G331R	10
c.1114C>G	p.P372A	P - TM6	P-TM interface	p.P372S	Similar consequences as pathogenic p.P372S	33
c.1115C>T	p.P372L					10
c.1307G>A	p.R436Q	C-linker (αB')	TM-(C-linker)-CNBD interface	p.R436W	Disturbed E467 interaction	11
c.1537G>A	p.G513R	CNBD (β2 - β3)	cNMP binding	p.G513E	Disturbed cNMP binding pocket	10
c.1537G>C	p.G513R					34
c.1573G>A	p.G525S	CNBD (β3 - β4)	TM-(C-linker)-CNBD interface	p.G525D	Disturbed oligomerization with other CNG channel subunits	35
c.1640T>G	p.F547C	CNBD (β6)	cNMP binding	p.F547L	Disturbance of direct interaction with cNMP	8
c.1705C>T	p.R569C	CNBD (β7)	cNMP binding	p.R569H	Disturbed cNMP binding pocket	10
c.1717T>C	p.Y573H	CNBD (β7 - β8)	TM-(C-linker)-CNBD interface	p.Y573C	Similar consequences as pathogenic p.Y573C	10

Table S4. Summary of missense VUS in *CNGA3* without overlapping pathogenic variants

Abbreviations: N-Term. (N-terminal region); TM (transmembrane domain); P (pore-gating region); CNBD (cyclic nucleotide binding domain); cNMP (cyclic nucleotide monophosphate). Colors correspond to Figure 4A.

Genotype	Proteotype	Location	Structure-function group	Potential molecular consequence of patient variant	Variant reference
c.479T>G	p.V160G	N-Term.	TM domain conformation	Loss of L239 interaction	10
c.513G>T	p.W171C	TM1	TM domain conformation	Loss of key hydrophobic interaction at hydrophobic core	11
c.536T>A	p.V179D	TM1	TM domain conformation	Physical and chemical disturbance at hydrophobic core	36
c.544A>G	p.N182D	TM1	TM domain conformation	Disturbed α-helical bundle packing	10
c.544A>T	p.N182Y	TM1	TM domain conformation	Disturbed α-helical bundle packing	14
c.556C>T	p.L186F	TM1	TM domain conformation	Physical disturbance at hydrophobic core	14
c.566G>A	p.R189K	TM1 - TM2	TM domain conformation	Loss of D207 interaction	10
c.572G>A	p.C191Y	TM1 - TM2	P-TM interface	Loss of key hydrophobic interaction at hydrophobic core	14
c.609G>T	p.W203C	TM2	TM domain conformation	Loss of L185 interaction	10
c.633T>A	p.D211E	TM2	TM domain conformation	Disturbed α-helical bundle packing	7
c.664G>C	p.A222P	TM2	TM domain conformation	Disturbed α-helix conformation	30
c.670A>G	p.T224A	TM2 - TM3	TM domain conformation	Disturbed α-helical bundle packing	10
c.671C>G	p.T224R	TM2 - TM3	TM domain conformation	Disturbed α-helical bundle packing	14
c.671C>T	p.T224I	TM2 - TM3	TM domain conformation	Disturbed α-helical bundle packing	11
c.704A>T	p.D235V	TM2 - TM3	.	Inconclusive	22
c.734C>T	p.T245M	TM2 - TM3	.	Inconclusive	16
c.740C>T	p.T247M	TM3	.	Inconclusive	11
c.743A>G	p.Q248R	TM3	TM domain conformation	Disturbed α-helical bundle packing (Loss of S282 interaction)	10
c.746T>C	p.F249S	TM3	TM domain conformation	Loss of W240 interaction	12
c.754G>A	p.D252N	TM3	TM domain conformation	Loss of K280 interaction	29
c.755A>C	p.D252A	TM3	TM domain conformation		13

c.772C>G	p.P258A	TM3	TM domain conformation	Disturbed α-helix conformation	10
c.773C>G	p.P258R	TM3	TM domain conformation		11
c.784G>C	p.A262P	TM3	TM domain conformation	Disturbed α-helix conformation	37
c.787T>G	p.Y263D	TM3	TM domain conformation	Disturbed α-helical bundle packing (Same consequence as our patient variant p.Q196L)	12
c.796G>A	p.V266M	TM3 - TM4	TM domain conformation	Disturbed α-helical bundle packing	38
c.811C>A	p.P271T	TM3 - TM4	TM domain conformation	Disturbed α-helix conformation	10
c.811C>G	p.P271A	TM3 - TM4	TM domain conformation		8
c.811C>T	p.P271S	TM3 - TM4	TM domain conformation		39
c.827A>G	p.N276S	TM4	TM domain conformation	Loss of S255 interaction	40
c.833T>C	p.L278P	TM4	TM domain conformation	Disturbed α-helix conformation	11
c.904A>G	p.R302G	TM5	TM domain conformation	Loss of E292 interaction	8
c.945C>G	p.H315Q	TM5	.	Inconclusive	41
c.952G>A	p.A318T	TM5	P-TM interface	Physical and chemical disturbance at hydrophobic core	42
c.965T>C	p.F322S	TM5	P-TM interface	Loss of key hydrophobic interaction at hydrophobic core (Loss of F330 interaction)	11
c.983T>C	p.I328T	TM5	P-TM interface	Loss of key hydrophobic interaction at hydrophobic core (Loss of W335 and V336 interaction)	10
c.989T>C	p.F330S	TM5 - P	P-TM interface	Loss of key hydrophobic interaction at hydrophobic core (Loss of F322 interaction)	11
c.1001C>T	p.S334F	TM5 - P	.	Inconclusive	15
c.1006G>T	p.V336F	TM5 - P	P-TM interface	Physical disturbance at hydrophobic core	7
c.1039C>T	p.R347C	TM5 - P	P-TM interface	Loss of E344 interaction	13
c.1058T>C	p.I353T	P	P-TM interface	Disturbed oligomerization with other CNG channel subunits	7
c.1061A>G	p.Y354C	P	P-TM interface	Loss of key hydrophobic interaction at hydrophobic core	10
c.1063A>G	p.S355G	P	.	Inconclusive	10
c.1070A>G	p.Y357C	P	P-TM interface	Loss of key hydrophobic interaction at hydrophobic core	43

c.1076C>T	p.S359F	P	P-TM interface	Disturbed α-helical bundle packing	10
c.1117G>A	p.V373M	P - TM6	P-TM interface	Disturbed α-helical bundle packing	10
c.1124A>G	p.D375G	TM6	P-TM interface	Disturbed oligomerization with other CNG channel subunits	13
c.1163G>A	p.G388D	TM6	.	Physical and chemical disturbance at hydrophobic core	13
c.1163G>T	p.G388V	TM6	.		13
c.1190G>T	p.G397V	TM6	Channel formation	Protrusion into channel	44
c.1201T>C	p.S401P	TM6	Channel formation	Disturbed α-helix conformation	12
c.1217T>C	p.M406T	TM6	.	Inconclusive	14
c.1243G>C	p.A415P	C-linker (αA')	.	Disturbed α-helix conformation	45
c.1255T>C	p.S419P	C-linker (αA')	.	Disturbed α-helix conformation	22
c.1256C>T	p.S419F	C-linker (αA')	.	Disturbed α-helical bundle packing	41
c.1286T>C	p.V429A	C-linker (αA' - αB')	TM-(C-linker)-CNBD interface	Loss of key hydrophobic interaction at hydrophobic core	39
c.1294G>T	p.D432Y	C-linker (αB')	TM-(C-linker)-CNBD interface	Disturbed oligomerization with other CNG channel subunits	13
c.1319G>C	p.W440S	C-linker (αB')	TM-(C-linker)-CNBD interface	Disturbed oligomerization with other CNG channel subunits	39
c.1343A>G	p.K448R	C-linker (αB' - αC')	.	Inconclusive	10
c.1391T>G	p.L464R	C-linker (αD')	TM-(C-linker)-CNBD interface	Disturbed oligomerization with other CNG channel subunits	46
c.1412A>G	p.N471S	C-linker (αD')	TM-(C-linker)-CNBD interface	Disturbed oligomerization with other CNG channel subunits	14
c.1435_1436delinsGT	p.K479V	C-linker (αE')	cNMP binding	Loss of G544 interaction	13
c.1454A>T	p.D485V	C-linker (αF')	cNMP binding	Disturbed cNMP binding pocket	14
c.1457G>A	p.C486Y	C-linker αF' - CNBD αA	cNMP binding	Disturbed cNMP binding pocket	47
c.1466G>T	p.G489V	CNBD (αA)	cNMP binding	Disturbed cNMP binding pocket	10
c.1513C>G	p.P505A	CNBD (β1 - β2)	TM-(C-linker)-CNBD interface	Disturbed loop conformation	34
c.1520A>G	p.D507G	CNBD (β1 - β2)	TM-(C-linker)-CNBD interface	Loss of R436 interaction (Same consequence as pathogenic p.R436W)	48

c.1535A>T	p.K512M	CNBD (β2 - β3)	cNMP binding	Disturbed cNMP binding pocket	49
c.1540G>A	p.D514N	CNBD (β2 - β3)	cNMP binding	Disturbance of cNMP binding pocket / Disturbed oligomerization with other CNG channel subunits	50
c.1541A>T	p.D514V	CNBD (β2 - β3)	cNMP binding	Disturbance of cNMP binding pocket / Disturbed oligomerization with other CNG channel subunits	25
c.1556T>C	p.M519T	CNBD (β3)	cNMP binding	Disturbance of direct interaction with cNMP	34
c.1557G>A	p.M519I	CNBD (β3)	cNMP binding	Disturbance of direct interaction with cNMP	51
c.1565T>C	p.I522T	CNBD (β3)	cNMP binding	Disturbed cNMP binding pocket (Loss of L527 interaction)	14
c.1579C>A	p.L527M	CNBD (β4)	cNMP binding	Disturbed cNMP binding pocket (Loss of I522 interaction)	52
c.1580T>G	p.L527R	CNBD (β4)	cNMP binding		48
c.1615G>A	p.V539M	CNBD (β5)	cNMP binding	Disturbed cNMP binding pocket	10
c.1618G>A	p.V540I	CNBD (β5)	.	Inconclusive	38
c.1621C>T	p.L541F	CNBD (β5)	cNMP binding	Disturbed cNMP binding pocket	46
c.1658T>A	p.L553Q	CNBD (β6 - β7)	cNMP binding	Disturbed cNMP binding pocket	10
c.1682G>A	p.G561E	CNBD (β6 - β7)	cNMP binding	Disturbed cNMP binding pocket	37
c.1708A>G	p.S570G	CNBD (β7)	TM-(C-linker)-CNBD interface	Loss of S574 interaction	10
c.1709G>A	p.S570N	CNBD (β7)	TM-(C-linker)-CNBD interface	Loss of S574 interaction	11
c.1709G>T	p.S570I	CNBD (β7)	TM-(C-linker)-CNBD interface	Loss of S574 interaction	38
c.1712T>C	p.I571T	CNBD (β7 - β8)	TM-(C-linker)-CNBD interface	Loss of L541 interaction	53
c.1736T>G	p.L579R	CNBD (β8)	cNMP binding	Disturbed cNMP binding pocket	10
c.1771T>C	p.Y591H	CNBD (αB – αC)	cNMP binding	Disturbance of direct interaction with cNMP	10
c.1775C>T	p.P592L	CNBD (αB – αC)	cNMP binding	Disturbance of direct interaction with cNMP	10
c.1793T>G	p.L598R	CNBD (αC)	cNMP binding	Disturbed cNMP binding pocket	13
c.1805G>A	p.G602E	CNBD (αC)	cNMP binding	Disturbed cNMP binding pocket	39

Supplemental References

1. Sergouniotis PI, Davidson AE, Mackay DS, et al. Recessive mutations in KCNJ13, encoding an inwardly rectifying potassium channel subunit, cause leber congenital amaurosis. Am J Hum Genet 2011;89(1):183-90. (In eng). DOI: 10.1016/j.ajhg.2011.06.002.
2. Hejtmancik JF, Jiao X, Li A, et al. Mutations in KCNJ13 cause autosomal-dominant snowflake vitreoretinal degeneration. Am J Hum Genet 2008;82(1):174-80. (In eng). DOI: 10.1016/j.ajhg.2007.08.002.
3. Abdelkader E, Enani L, Schatz P, Safieh L. Severe retinal degeneration at an early age in Usher syndrome type 1B associated with homozygous splice site mutations in MYO7A gene. Saudi J Ophthalmol 2018;32(2):119-125. (In eng). DOI: 10.1016/j.sjopt.2017.10.004.
4. Meena R, Ayub M. Genetics Of Human Hereditary Hearing Impairment. J Ayub Med Coll Abbottabad 2017;29(4):671-676. (In eng).
5. Ashkenazy H, Abadi S, Martz E, et al. ConSurf 2016: an improved methodology to estimate and visualize evolutionary conservation in macromolecules. Nucleic Acids Res 2016;44(W1):W344-50. (In eng). DOI: 10.1093/nar/gkw408.
6. Zheng X, Hu Z, Li H, Yang J. Structure of the human cone photoreceptor cyclic nucleotide-gated channel. Nat Struct Mol Biol 2022;29(1):40-46. (In eng). DOI: 10.1038/s41594-021-00699-y.
7. Liang X, Dong F, Li H, Li H, Yang L, Sui R. Novel CNGA3 mutations in Chinese patients with achromatopsia. Br J Ophthalmol 2015;99(4):571-6. (In eng). DOI: 10.1136/bjophthalmol-2014-305432.
8. Zelinger L, Cideciyan AV, Kohl S, et al. Genetics and Disease Expression in the CNGA3 Form of Achromatopsia: Steps on the Path to Gene Therapy. Ophthalmology 2015;122(5):997-1007. (In eng). DOI: 10.1016/j.ophtha.2014.11.025.
9. Ezquerra-Inchausti M, Anasagasti A, Barandika O, et al. A new approach based on targeted pooled DNA sequencing identifies novel mutations in patients with Inherited Retinal Dystrophies. Scientific Reports 2018;8(1):15457. DOI: 10.1038/s41598-018-33810-3.
10. Solaki M, Baumann B, Reuter P, et al. Comprehensive variant spectrum of the CNGA3 gene in patients affected by achromatopsia. Hum Mutat 2022;43(7):832-858. (In eng). DOI: 10.1002/humu.24371.
11. Li S, Huang L, Xiao X, Jia X, Guo X, Zhang Q. Identification of CNGA3 mutations in 46 families: common cause of achromatopsia and cone-rod dystrophies in Chinese patients. JAMA Ophthalmol 2014;132(9):1076-83. (In eng). DOI: 10.1001/jamaophthalmol.2014.1032.
12. Nishiguchi KM, Sandberg MA, Gorji N, Berson EL, Dryja TP. Cone cGMP-gated channel mutations and clinical findings in patients with achromatopsia, macular degeneration, and other hereditary cone diseases. Hum Mutat 2005;25(3):248-58. (In eng). DOI: 10.1002/humu.20142.
13. Sun W, Li S, Xiao X, Wang P, Zhang Q. Genotypes and phenotypes of genes associated with achromatopsia: A reference for clinical genetic testing. Mol Vis 2020;26:588-602. (In eng).
14. Wissinger B, Gamer D, Jägle H, et al. CNGA3 Mutations in Hereditary Cone Photoreceptor Disorders. The American Journal of Human Genetics 2001;69(4):722-737. DOI: 10.1086/323613.

15. Sundaram V, Wilde C, Aboshiha J, et al. Retinal structure and function in achromatopsia: implications for gene therapy. Ophthalmology 2014;121(1):234-245. (In eng). DOI: 10.1016/j.ophtha.2013.08.017.
16. Johnson S, Michaelides M, Aligianis IA, et al. Achromatopsia caused by novel mutations in both CNGA3 and CNGB3. J Med Genet 2004;41(2):e20. (In eng). DOI: 10.1136/jmg.2003.011437.
17. Burgueño-Montañés C, Colunga Cueva M, Costales Álvarez C. [A novel mutation in the CNGA3 gene responsible for incomplete achromatopsia]. Arch Soc Esp Oftalmol 2014;89(3):107-9. (In spa). DOI: 10.1016/j.oftal.2012.07.019.
18. Holtan JP, Selmer KK, Heimdal KR, Bragadóttir R. Inherited retinal disease in Norway - a characterization of current clinical and genetic knowledge. Acta Ophthalmol 2020;98(3):286-295. (In eng). DOI: 10.1111/aos.14218.
19. Taylor RL, Parry NRA, Barton SJ, et al. Panel-Based Clinical Genetic Testing in 85 Children with Inherited Retinal Disease. Ophthalmology 2017;124(7):985-991. (In eng). DOI: 10.1016/j.ophtha.2017.02.005.
20. Wiszniewski W, Lewis RA, Lupski JR. Achromatopsia: the CNGB3 p.T383fsX mutation results from a founder effect and is responsible for the visual phenotype in the original report of uniparental disomy 14. Hum Genet 2007;121(3-4):433-9. (In eng). DOI: 10.1007/s00439-006-0314-y.
21. Kohl S, Marx T, Giddings I, et al. Total colourblindness is caused by mutations in the gene encoding the alpha-subunit of the cone photoreceptor cGMP-gated cation channel. Nat Genet 1998;19(3):257-9. (In eng). DOI: 10.1038/935.
22. Weisschuh N, Obermaier CD, Battke F, et al. Genetic architecture of inherited retinal degeneration in Germany: A large cohort study from a single diagnostic center over a 9-year period. Hum Mutat 2020;41(9):1514-1527. (In eng). DOI: 10.1002/humu.24064.
23. Shaikh RS, Reuter P, Sisk RA, et al. Homozygous missense variant in the human CNGA3 channel causes cone-rod dystrophy. Eur J Hum Genet 2015;23(4):473-80. (In eng). DOI: 10.1038/ejhg.2014.136.
24. Li FF, Huang XF, Chen J, et al. Identification of novel mutations by targeted exome sequencing and the genotype-phenotype assessment of patients with achromatopsia. J Transl Med 2015;13:334. (In eng). DOI: 10.1186/s12967-015-0694-7.
25. Genead MA, Fishman GA, Rha J, et al. Photoreceptor structure and function in patients with congenital achromatopsia. Invest Ophthalmol Vis Sci 2011;52(10):7298-308. (In eng). DOI: 10.1167/iovs.11-7762.
26. Saqib MA, Nikopoulos K, Ullah E, et al. Homozygosity mapping reveals novel and known mutations in Pakistani families with inherited retinal dystrophies. Sci Rep 2015;5:9965. (In eng). DOI: 10.1038/srep09965.
27. Koeppen K, Reuter P, Ladewig T, et al. Dissecting the pathogenic mechanisms of mutations in the pore region of the human cone photoreceptor cyclic nucleotide-gated channel. Hum Mutat 2010;31(7):830-9. (In eng). DOI: 10.1002/humu.21283.

28. Kuniyoshi K, Muraki-Oda S, Ueyama H, et al. Novel mutations in the gene for α-subunit of retinal cone cyclic nucleotide-gated channels in a Japanese patient with congenital achromatopsia. Jpn J Ophthalmol 2016;60(3):187-97. (In eng). DOI: 10.1007/s10384-016-0424-6.
29. Koeppen K, Reuter P, Kohl S, Baumann B, Ladewig T, Wissinger B. Functional analysis of human CNGA3 mutations associated with colour blindness suggests impaired surface expression of channel mutants A3(R427C) and A3(R563C). Eur J Neurosci 2008;27(9):2391-401. (In eng). DOI: 10.1111/j.1460-9568.2008.06195.x.
30. Reuter P, Koeppen K, Ladewig T, Kohl S, Baumann B, Wissinger B. Mutations in CNGA3 impair trafficking or function of cone cyclic nucleotide-gated channels, resulting in achromatopsia. Hum Mutat 2008;29(10):1228-36. (In eng). DOI: 10.1002/humu.20790.
31. Azam M, Collin RW, Shah ST, et al. Novel CNGA3 and CNGB3 mutations in two Pakistani families with achromatopsia. Mol Vis 2010;16:774-81. (In eng).
32. Carss KJ, Arno G, Erwood M, et al. Comprehensive Rare Variant Analysis via Whole-Genome Sequencing to Determine the Molecular Pathology of Inherited Retinal Disease. Am J Hum Genet 2017;100(1):75-90. (In eng). DOI: 10.1016/j.ajhg.2016.12.003.
33. Sharon D, Ben-Yosef T, Goldenberg-Cohen N, et al. A nationwide genetic analysis of inherited retinal diseases in Israel as assessed by the Israeli inherited retinal disease consortium (IIRDC). Hum Mutat 2020;41(1):140-149. (In eng). DOI: 10.1002/humu.23903.
34. Huang L, Xiao X, Li S, et al. Molecular genetics of cone-rod dystrophy in Chinese patients: New data from 61 probands and mutation overview of 163 probands. Exp Eye Res 2016;146:252-258. (In eng). DOI: 10.1016/j.exer.2016.03.015.
35. Patel N, Aldahmesh MA, Alkuraya H, et al. Expanding the clinical, allelic, and locus heterogeneity of retinal dystrophies. Genet Med 2016;18(6):554-62. (In eng). DOI: 10.1038/gim.2015.127.
36. Dubis AM, Cooper RF, Aboshiha J, et al. Genotype-dependent variability in residual cone structure in achromatopsia: toward developing metrics for assessing cone health. Invest Ophthalmol Vis Sci 2014;55(11):7303-11. (In eng). DOI: 10.1167/iovs.14-14225.
37. Zobor D, Werner A, Stanzial F, et al. The Clinical Phenotype of CNGA3-Related Achromatopsia: Pretreatment Characterization in Preparation of a Gene Replacement Therapy Trial. Invest Ophthalmol Vis Sci 2017;58(2):821-832. (In eng). DOI: 10.1167/iovs.16-20427.
38. Thiadens AA, Roosing S, Collin RW, et al. Comprehensive analysis of the achromatopsia genes CNGA3 and CNGB3 in progressive cone dystrophy. Ophthalmology 2010;117(4):825-30.e1. (In eng). DOI: 10.1016/j.ophtha.2009.09.008.
39. Georgiou M, Litts KM, Kalitzeos A, et al. Adaptive Optics Retinal Imaging in CNGA3-Associated Achromatopsia: Retinal Characterization, Interocular Symmetry, and Intrafamilial Variability. Investigative Ophthalmology & Visual Science 2019;60(1):383-396. DOI: 10.1167/iovs.18-25880.

40. Saqib MA, Awan BM, Sarfraz M, Khan MN, Rashid S, Ansar M. Genetic analysis of four Pakistani families with achromatopsia and a novel S4 motif mutation of CNGA3. Jpn J Ophthalmol 2011;55(6):676-80. (In eng). DOI: 10.1007/s10384-011-0070-y.
41. Patel A, Hayward JD, Tailor V, et al. The Oculome Panel Test: Next-Generation Sequencing to Diagnose a Diverse Range of Genetic Developmental Eye Disorders. Ophthalmology 2019;126(6):888-907. (In eng). DOI: 10.1016/j.ophtha.2018.12.050.
42. Li L, Chen Y, Jiao X, et al. Homozygosity Mapping and Genetic Analysis of Autosomal Recessive Retinal Dystrophies in 144 Consanguineous Pakistani Families. Invest Ophthalmol Vis Sci 2017;58(4):2218-2238. (In eng). DOI: 10.1167/iovs.17-21424.
43. Vincent A, Wright T, Billingsley G, Westall C, Héon E. Oligocone trichromacy is part of the spectrum of CNGA3-related cone system disorders. Ophthalmic Genet 2011;32(2):107-13. (In eng). DOI: 10.3109/13816810.2010.544366.
44. Ahuja Y, Kohl S, Traboulsi EI. CNGA3 mutations in two United Arab Emirates families with achromatopsia. Mol Vis 2008;14:1293-7. (In eng).
45. Stone EM, Andorf JL, Whitmore SS, et al. Clinically Focused Molecular Investigation of 1000 Consecutive Families with Inherited Retinal Disease. Ophthalmology 2017;124(9):1314-1331. (In eng). DOI: 10.1016/j.ophtha.2017.04.008.
46. Greenberg JP, Sherman J, Zweifel SA, et al. Spectral-domain optical coherence tomography staging and autofluorescence imaging in achromatopsia. JAMA Ophthalmol 2014;132(4):437-45. (In eng). DOI: 10.1001/jamaophthalmol.2013.7987.
47. Abouelhoda M, Sobahy T, El-Kalioby M, et al. Clinical genomics can facilitate countrywide estimation of autosomal recessive disease burden. Genet Med 2016;18(12):1244-1249. (In eng). DOI: 10.1038/gim.2016.37.
48. Lam K, Guo H, Wilson GA, Kohl S, Wong F. Identification of variants in CNGA3 as cause for achromatopsia by exome sequencing of a single patient. Arch Ophthalmol 2011;129(9):1212-7. DOI: 10.1001/archophthalmol.2011.254.
49. Carrigan M, Duignan E, Malone CP, et al. Panel-Based Population Next-Generation Sequencing for Inherited Retinal Degenerations. Sci Rep 2016;6:33248. (In eng). DOI: 10.1038/srep33248.
50. Arshad MW, Lee Y, Malik MA, et al. Identification of Novel Mutation in CNGA3 gene by Whole-Exome Sequencing and In-Silico Analyses for Genotype-Phenotype Assessment with Autosomal Recessive Achromatopsia in Pakistani families. J Pak Med Assoc 2019;69(2):183-189. (In eng).
51. Ellingford JM, Barton S, Bhaskar S, et al. Molecular findings from 537 individuals with inherited retinal disease. J Med Genet 2016;53(11):761-767. (In eng). DOI: 10.1136/jmedgenet-2016-103837.
52. Wang X, Wang H, Cao M, et al. Whole-exome sequencing identifies ALMS1, IQCB1, CNGA3, and MYO7A mutations in patients with Leber congenital amaurosis. Hum Mutat 2011;32(12):1450-9. (In eng). DOI: 10.1002/humu.21587.

53. Jinda W, Tuekprakhon A, Thongnoppakhun W, Limwongse C, Trinavarat A, Atchaneeyasakul LO. Molecular and clinical characterization of Thai patients with achromatopsia: identification of three novel disease-associated variants in the CNGA3 and CNGB3 genes. Int Ophthalmol 2021;41(1):121-134. (In eng). DOI: 10.1007/s10792-020-01559-2.
